# Dihydropyridine Enhances the Antioxidant Capacities of Lactating Dairy Cows under Heat Stress Condition

**DOI:** 10.3390/ani10101812

**Published:** 2020-10-05

**Authors:** Meng-Fei Yu, Xin-Mao Zhao, Hang Cai, Jian-Ming Yi, Guo-Hua Hua

**Affiliations:** 1 Hubei Provincial Key Laboratory for Protection and Application of Special Plant Germplasm in Wuling Area of China, College of Life Sciences, South-Central University for Nationalities, 182 Min Zu Da Dao, Wuhan 430074, China; 2011093@mail.scuec.edu.cn; 2 Key Laboratory of Agricultural Animal Genetic, Breeding, and Reproduction for Ministry of Education, College of Animal Science and Technology, Huazhong Agricultural University, Wuhan 430070, China; alabaster1001@163.com (X.-M.Z.); caihang1001@126.com (H.C.)

**Keywords:** antioxidation, dihydropyridine, dairy cow, heat stress, ruminal bacterial community

## Abstract

**Simple Summary:**

Additives contribute to improving the health of dairy cows, enhancing antioxidative capacities, and/or increasing milk production, etc. To alleviate the harmful effects of heat stress on dairy cows, a few feed additives studies have been conducted. Dihydropyridine has been used as a feed additive in dairy cow diets. However, the underlying mechanisms of its beneficial effects still remain unclear. In the present study, dairy cows were randomly divided into a control group and a dihydropyridine treatment group under heat stress in summer. The rumen and blood samples of dairy cows were collected to determine the changes in their antioxidative capacities. Meanwhile, the effects of dihydropyridine on ruminal microbial communities were also analyzed. Our data demonstrated that dihydropyridine enhanced the antioxidative capacities of dairy cows under heat stress conditions.

**Abstract:**

Heat stress (HS), a nonspecific response to environmental heat, can seriously affect dairy cow health. Feed additives may alleviate HS in dairy cows by improving rumen fermentation efficacy, stimulating feed consumption, enhancing vasodilation, and/or improving antioxidant capacity. The temperature–humidity index (THI) indicates that spring is a non-HS season, and summer is an HS season. HS results in the decrease in dairy cow antioxidant capacities. Our results indicated the decrease in superoxide dismutase (SOD), glutathione peroxidase (GSH-Px), catalase (CAT), and total antioxidation (T-AOC) levels and the increase in malondialdehyde (MDA) level during HS season. Meanwhile, antioxidant indexes (SOD, GSH-Px, and T-AOC) were positively correlated with milk yield (*p* < 0.01), whereas MDA exhibited a significant negative correlation with milk yield (*p* < 0.05). In addition, the effects of dihydropyridine (DHP) on antioxidant capacity and ruminal microbial communities in dairy cows under HS were investigated. During summer, dairy cows were randomly assigned into two groups under HS, including a standard diet (S-ND) group and standard diet with 3 g/day/cow DHP (S-D) group. DHP treatment significantly restored SOD and GSH-Px levels under HS. Denaturing gradient gel electrophoresis results indicated that the DHP altered ruminal bacterial community mainly composed Proteobacteria and Firmicutes in dairy cows under HS. Our results suggest that DHP can enhance the antioxidant abilities of dairy cows with favorable effects on ruminal microbial communities under HS, further alleviating HS on dairy cows.

## 1. Introduction

Stress is the physical responses of animals to external and internal stimuli. Various stresses, such as oxidation and heat, can result in serious stress responses in dairy cows. For example, the primary reason for reproductive diseases such as retained placenta, endometritis, acute/chronic mastitis was oxidative stress [1]. Heat stress (HS) in dairy cows is a nonspecific response to the hot and humid environment, which exerts adverse impacts on the performance and physiological functions of the cows [2,3]. Lactating cows are more sensitive to the HS, which is attributed to their physiological characteristics and special nutritional requirements [4]. In turn, HS can stimulate the release of endocrine hormones which can damage the function of the immune system and increase morbidity. HS can cause serious problems, such as health damage and milk yield decrease.

Daily metabolic activities in dairy cows produce a large number of free radicals and other bioactive substances [5]. The production and elimination of these radicals and bioactive substances are kept in a balanced status by the antioxidant systems of the dairy cows. This balance can be interrupted by many detrimental factors such as diseases, oxidative stress, HS etc. The interrupted balance can be monitored by measuring the antioxidative capacities of serum and rumen fluids. The antioxidant capacity of dairy cows is usually evaluated by five parameters, namely, total antioxidation (T-AOC), superoxide dismutase (SOD), Glutathione peroxidase (GSH-Px), catalase (CAT), and malondialdehyde (MDA), which refer to the abilities of total antioxidation, eliminating free radicals, cleaning up peroxide, eliminating H_2_O_2_, and ability of lipid peroxidation, respectively. Among them, SOD, GSH-Px, and CAT function coordinately to eradicate the intracellular harmful substances and protect the cells. HS can interrupt the expressions and functions of these enzymes, which in turn results in the accumulation of superoxide anion radicals. The increased superoxide anion radicals lead to the formation of MDA which induces cell death by breaking DNA. In general, relatively high levels of T-AOC, SOD, GSH-Px, and CAT, and a relatively low value of MDA represent a better antioxidant capacity in dairy cows. These five antioxidant indexes in blood and/or ruminal fluids are usually used to evaluate the stress status of dairy cows [6].

Dihydropyridine (DHP) is a type of Ca^2+^ channel antagonist, which can inhibit Ca^2+^ influx into the cytoplasm and decrease the concentration of cytoplasmic Ca^2+^. DHP is widely used as a therapeutic agent for diseases such as myocardial ischemia [7], hypertension [8], and renal diseases [9]. Additionally, DHP has been used as an additive in animal diets due to its antioxidant ability to protect oil, vitamin A, beta-carotene from oxidation [10]. Meanwhile, DHP can alter the levels of hormones in the serum, which is beneficial for the promotion of the growth of animals, enhancing reproductive ability, and increasing milking capacity. DHP can be metabolized without toxic effects in many species, such as pigs and chickens [11,12]. These evidences indicate DHP is a good feed additive candidate for alleviating the harmful responses induced by HS. However, the effects of DHP diet on ruminal antioxidant capacity and the underlying mechanisms remain unclear.

In this study, we investigated the effects of DHP diet on the alteration in the structure and components of ruminal microbes (bacteria, protozoa, archaea, and fungi) in lactating dairy cows. Moreover, the effects of DHP on the antioxidant capacity, such as SOD, GSH-Px, T-AOC, and MDA in the serum and ruminal fluids of dairy cows were also analyzed. The elucidation of the effects of DHP in dairy cows will be helpful in improving cows’ health and production performance under HS.

## 2. Materials and Methods

### 2.1. Animals and Ethical Statement

All animal experiments were approved by the Huazhong Agricultural University animal care and use committee. All experimental protocols were also approved by the committee mentioned above and performed in accordance with the relevant guidelines and regulations (Approval No: YJM-201001).

### 2.2. Determination of Anti-Oxidative Indexes

To determine anti-oxidative indexes under non-HS and HS conditions, a total of forty healthy Chinese Holstein cows were chosen. These dairy cows were all in the mid-lactation (124.8 ± 13.4 days) period with similar parity (2.8 ± 0.7), body weight (513.0 ± 6.0 kg) and milking abilities (37.1 ± 2.5 kg). All cows were fed with standard diet from April 1st to July 14th. The standard diets were formulated to meet the feeding standards of China Holstein cows (NY/T 34-2004, Table 1). All cows were provided with ad libitium intake at 12-h intervals to meet nutritional requirements. On April 15th and July 14th, blood samples were collected from all dairy cows. Five antioxidant indexes including SOD, GSH-Px, T-AOC, MDA, and CAT were detected from the serum samples. Meanwhile, the correlations between antioxidant indexes and milk yield were analyzed.

### 2.3. Detection of HS Using Temperature–Humidity Index (THI) Method

Dairy cows were housed in a semi-open barn with ventilation fans and without sprinkler facilities. The HS level of these dairy cows were evaluated by monitoring the changes in ambient temperature and humidity using six dry-wet bulb hygrometers (Model, TAL-2, Beijing Kawe Meters Co., Ltd., Beijing, China). Three hygrometers were hung 2 m above the aisle and the beds indoors, while the other three hygrometers were hung outdoors. During the experimental period, the values were recorded at 08:00, 14:00, and 20:00. THI was calculated using the following formula: THI = 0.72 × (Td + Tw) + 40.6, where Td and Tw indicated dry bulb temperature and wet bulb temperature in °C, respectively [13].

### 2.4. DHP Supplement Experiment

Compared to the milk yield in April, the cows with milk yield decreased >50% in July were considered as heat-sensitive cows. To evaluate the effects of DHP on the antioxidant capacities and ruminal microbes in mid-lactation dairy cows, the selected twenty heat-sensitive cows were randomly divided into two groups: a control group and an experimental group (*n* = 10). The cows in the control group (S-ND) were fed with the standard diet (Table 1), while the cows in the experimental group (S-D) were fed with the standard diet containing 3 g/day/cow DHP from 15 July to 30 July according to the Veterinary Drug Quality Standards 2003 edition [14]. DHP was first mixed with a small amount of concentrate, followed by an expanding mixture with all other feed. The effects of DHP on the antioxidant capacities and the composition of ruminal microbes in the dairy cows were analyzed. The blood samples were collected on 30 July for the determination of antioxidant indexes (SOD, GSH-Px, T-AOC, MDA, and CAT). In addition, ruminal fluids were collected on 30 July and 31 July and stored at −20 °C for the analysis of the composition of ruminal microbes.

### 2.5. Blood Sample Collection

On July 30, whole blood was collected as previously described with some modifications [15]. Ten milliliters of whole blood was collected at 10:00 a.m. via tail vein. After being kept on ice for 30 min, the blood was centrifuged at 5000 rpm for 5 min to isolate the serum. The obtained serum was stored at −20 °C, for subsequent experiments.

### 2.6. Ruminal Fluids Collection

Ruminal fluids were collected as previously described elsewhere with some modifications [16,17]. Briefly, 30 mL ruminal fluids were collected using rumenocentesis. These ruminal fluids were collected at 13:00 on 30 July and 07:00 and 10:00 on 31 July and stored at −20 °C for further experiments. These samples were to be used to analyze the contents of SOD, GSH-Px, T-AOC, MDA, and CAT, in the blood and ruminal fluids, respectively.

### 2.7. Detection of Antioxidant Status of Dairy Cows

The contents of GSH-Px, SOD, CAT, T-AOC, and MDA in the serum and ruminal fluids were measured using five different kits, namely, the GSH-Px assay kit (Cat.: A005), SOD assay kit (Cat.: A001-3), T-AOC assay kit (Cat.: A015-2), CAT assay kit (Cat.: A007-1-1), and the MDA assay kit (Cat.: A003-1), according to the manufacturer’s instructions, respectively. All these kits were commercially purchased from by Nanjing Jiancheng Bioengineering Institute (Nanjing, China).

### 2.8. DNA Extraction

The genomic DNA of microbiota in the rumen fluids was extracted using a DNA isolation kit (Cat.: AU2001, Bioteke, Beijing, China) according to the manufacturer’s instructions. The purity and concentration were determined by a spectrophotometer (Nanodrop 2000, Thermo Scientific, Wilmington, DE, USA). The DNA samples were stored at −20 °C for the further processing.

### 2.9. Real-Time PCR

The genomic DNA mentioned above was used as the template to amplify the 16S rRNA V6-V8 region. All primers were synthesized by Sangon Biotech (Shanghai) Co., Ltd. (Shanghai, China). Detailed information of the primers (total bacteria, fungi, protozoa, Methanogens, *B. fibrisolvens, C.proteoclasticum*, *F. succinogenes*, *R. albus*, and *R. flavefaciens*) is shown in Table 2. The PCR system included 12.5 μL SYBR^®^ Green Real-Time PCR MasterMix (St. Louis, MO, USA), 0.5 μL forward primer (10 mM), 0.5 μL reverse primer (10 mM), 1 μL DNA template, and 9.5 μL sterile deionized water. The procedures for Real-time PCR were as follows: pre-denaturalization for 3 min at 95.0 °C, 40 cycles of denaturalization for 30 s at 95.0 °C, annealing for 30 s at 60.0 °C, and extension for 30 s at 72.0 °C, followed by extension for 7 min at 72.0 °C. The relative contents of the above-mentioned eight microbes in ruminal fluids were calculated using total bacteria as a reference, as previously described [18] or designed using NCBI primer-Blast (www.ncbi.nlm.nih.gov/tools/primer-blast/). 

### 2.10. Denaturing Gradient Gel Electrophoresis (DGGE)

A pair of general primers targeting bacterial V6-V8 regions were used to amplify the 16S rRNA of bacteria in ruminal fluids. The primers were: forward, 5′-CGC CCG GGG CGC GCC CCG GGC GGG GGG GCG GGG GCA CGG GGG GAA CGC GAA GAA CCT TAC-3′ (underline means GC clamp); reverse, 5′-CGG TGT GTA CAA GAC CC-3′. The procedures for PCR were as follows: pre-denaturalization for 10 min at 94.0 °C, 35 cycles of denaturalization for 45 s at 94.0 °C, annealing for 45 s at 62.8 °C, extension for 45 s at 72.0 °C, followed by extension for 10 min at 72.0 °C. 

Specific separation of PCR amplification products was performed by DGGE, as previously described [24]. Briefly, PCR-amplified samples were subjected to 7-h electrophoresis at 150 V through a 40% polyacrylamide gel at 60 °C, and then were subjected to electrophoresis again through 40% to 60% denaturing gradient gel. After two electrophoreses, the gel was stained with SYBR Green I (Molecular Probes, St. Louis, MO, USA) for 30 min with 1× Tris-acetate-EDTA (ethylenediaminetetraacetic acid) (TAE) buffer. Then the grey values of the bands in the gel were examined using Quantity One software (ChemiDoc XRS+, Bio-Rad laboratories, Hercules, CA, USA). The dominant bands were cut off using a sterile scalpel and immersed in deionized water for 5 h at 37 °C and 4 °C overnight, respectively. Then, one microliter of this sample was used as template to amplify the V6-V8 region of the 16S rDNA gene with PCR primers without GC clamps. After agarose electrophoresis, these PCR products were purified and inserted into pMD-18-T® plasmids. The recombinant pMD-18-T plasmids were transformed into DH5α ® component cells. These positive transformed cells were extracted using a plasmid mini-kit (TIANGEN prep Mini Plasmid Kit, Beijing, China) according to the manufacturer’s instructions.

### 2.11. DNA Sequencing and Phylogenetic Tree Analysis

The plasmid DNA mentioned above was sequenced by Invitrogen Biotechnology Co., Ltd. (Shanghai, China) and analyzed using the BLAST program (http://www.ncbi.nlm.nih.gov/blast) [24,25]. The sequence distance matrix for all pair wise sequence combinations was analyzed using MEGA 4.0.2 software with the neighbor-joining phylogenetic tree method. All 16S rRNA sequence reads have been submitted to the NCBI Genbank with the accession numbers of JF798509-JF798517.

### 2.12. Statistical Analysis

Data were expressed as LSMEANS (means obtained based on the Least Square Error Method) ± SEM. The normality of data distribution was first assessed using the Shapiro–Wilk test. When necessary, data were logarithmically, or Box–Cox transformed to achieve normality. The Pearson correlation coefficient was used to analyze the correlations between the antioxidant indexes and milk yield. Student’s t-test was performed to evaluate the differences of serum antioxidative indexes in non-HS and HS seasons and serum/ruminal antioxidative indexes between the control and experimental groups. *p* < 0.05 was considered statistically significant. All the statistical analyses were performed using the SAS 8.1 software (SAS Institute, Cary, NC, USA).

## 3. Results

### 3.1. THI Determination in Different Seasons

**Figure 1:**
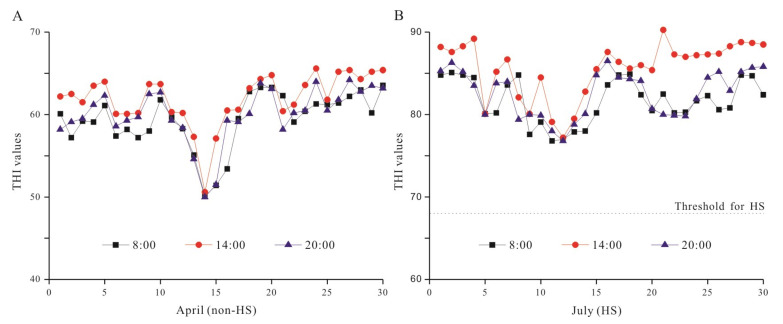
Temperature–humidity index (THI) values of the dairy barn in April and July. (**A**) Daily changes in THI values in the non-heat stress (HS) season (April); (**B**) Daily changes in THI values in the HS season (July). THI was recorded in the morning (08:00), afternoon (14:00), and night (20:00) each day. All the THI values were higher than the HS threshold of 68 in July.

To confirm the THI index in dairy farm in spring and summer, we recorded the THI in April and July (Figure 1). In April, the THI values ranged from 50.15 to 65.44. All the THI values were below the HS threshold of 68 [26], indicating the non-HS condition during April. In July, THI values varied from 76.96 to 90.28, and the average THI at afternoon was 83.24. All the THI values were higher than the HS threshold of 68. These data suggested that the dairy cows were under HS conditions in July.

### 3.2. Changes in Serum Antioxidant Capacity of Dairy Cows in Spring (Non-HS) and Summer (HS)

**Figure 2:**
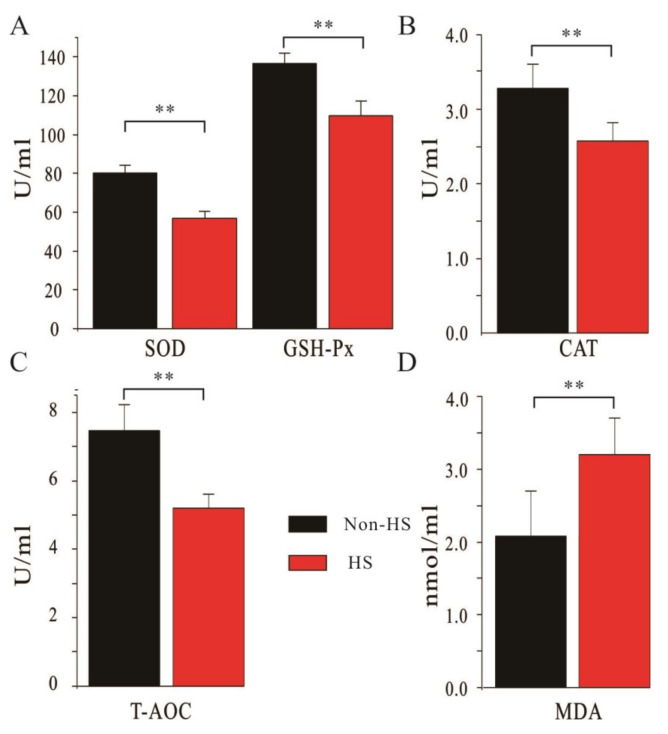
Serum antioxidant capacities of dairy cows in non-HS and HS season. All the 40 dairy cows were fed with standard diet. Blood samples were collected during spring (non-HS season) and summer (HS season), respectively. Superoxide dismutase (SOD) and glutathione peroxidase (GSH-Px) (**A**), catalase (CAT) (**B**), total antioxidation (T-AOC) (**C**), and malondialdehyde (MDA) (**D**) levels in the serum of dairy cows in non-HS and HS seasons. **: *p* < 0.01

The serum antioxidant indexes of dairy cows in the non-HS season and HS season are shown in Figure 2. The results indicated that the four antioxidant indexes (SOD, GSH-Px, CAT, and T-AOC) in dairy cows under HS conditions were all significantly lower than those in non-HS season (*p* < 0.05). Consistently, the content of MDA in dairy cows under summer HS conditions was significantly increased, compared with that under non-HS conditions (*p* < 0.05), suggesting the accumulation of superoxidative anion radicals. These results indicated the reduction in antioxidant capacity under HS. Thus, these five antioxidant indexes could be the useful candidates to indicate HS in dairy cows.

### 3.3. Correlation Analysis between the Serum Antioxidant Capacity and Milk Yield

SOD, GSH-Px, and T-AOC showed negative correlations with the decrease rate of milk yield (*p* < 0.01) (Table 3). Meanwhile, MDA showed a significant positive correlation with the decrease rate of milk yield (*p* < 0.05). However, CAT showed no significant difference with the decrease rate of milk yield (*p* > 0.05). Based on these data, SOD, GSH-Px, T-AOC, and MDA were selected to indicate the antioxidant capacities of dairy cows.

### 3.4. Effects of DHP on Serum Antioxidant Capacity of Dairy Cows under HS Conditions

**Figure 3:**
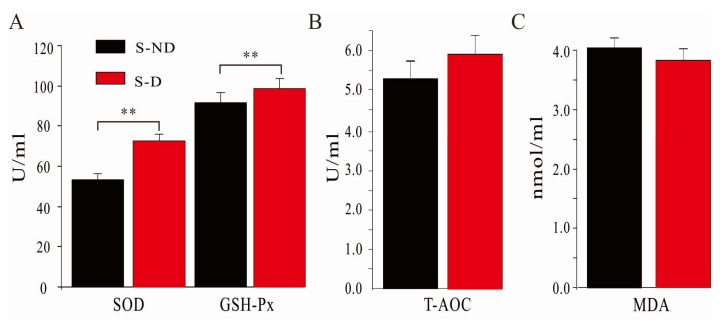
Effects of dihydropyridine (DHP) on serum antioxidative index. Twenty heat-sensitive dairy cows were divided into control (S-ND) group and experimental (S-D) group. The control group (S-ND) was fed with the standard diet. The experimental group (S-D) was fed with the standard diet containing 3 g/day/cow DHP. (**A**) Effects of DHP on serum SOD and GSH-Px concentration; (**B**) effects of DHP on serum T-AOC level; (**C**) effects of DHP on serum MDA content. **: *p* < 0.01.

The effects of DHP on the dairy cow serum antioxidant indexes under HS conditions are shown in Figure 3. The values of SOD and GSH-Px in the experiment (S-D) group were significantly increased, compared with those in the control (S-ND) group (*p* < 0.01). DHP treatment favorably increased T-AOC level and decreased MDA level, but the changes in these two indexes were not statistically significant (*p* > 0.05). These data indicated that the addition of DHP in diet might enhance the antioxidant capacity of dairy cows under HS conditions.

### 3.5. Effects of DHP on Ruminal Antioxidant Capacity of Dairy Cows under HS Condition

**Figure 4:**
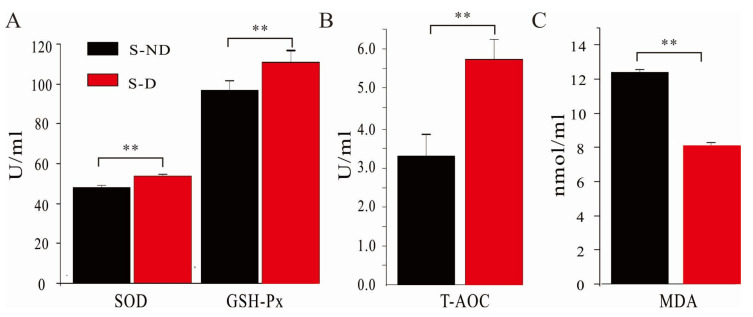
Effects of DHP on the ruminal antioxidative capacity of dairy cows under HS. Ruminal fluids were collected at 13:00 on 30 July and 07:00 and 10:00 on 31 July. (**A**) Effects of DHP on ruminal SOD and GSH-Px concentrations of dairy cows under HS; (**B**) effects of DHP on ruminal T-AOC level of dairy cows under HS; (**C**) effects of DHP on ruminal MDA concentration of dairy cows under HS. **: *p* < 0.01.

The impacts of DHP on the ruminal antioxidant status on dairy cow are presented in Figure 4. Our results indicated that the addition of DHP to the diet significantly increased the concentrations of SOD, GSH-Px, and T-AOC in the experimental group (*p* < 0.01). These changes mean an enhanced ability to eliminate free radicals, peroxide, and H2O2, thus alleviating the harmful effects of HS on the dairy cows. Similarly, the content of MDA, a toxic metabolic product, in the experimental group was significantly lower than that in the control group (*p* < 0.01), indicating an improved antioxidant capacity of dairy cows. Together, DHP addition in the diet significantly enhanced the ruminal antioxidant ability of dairy cows under HS conditions.

### 3.6. Effects of DHP on Ruminal Microbial Communities

**Figure 5:**
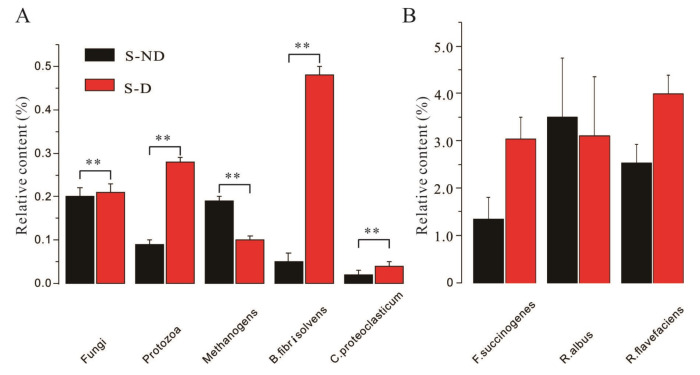
Effects of DHP on ruminal microbial communities in dairy cows. (**A**) Effects of DHP on the contents of fungi, protozoa, methanogens, *B. fibrisolvens*, and *C.proteoclasticum* in the rumen of dairy cows under HS conditions; (**B**) effects of DHP on the relative contents of *F. succinogenes*, *R. albus*, and *R. flavefaciens* in the rumen of dairy cows under HS conditions. **: *p* < 0.01

The effects of DHP on ruminal microbial communities are shown in Figure 5. The contents of fungi, protozoa, *B. fibrisolvens*, and *C.proteoclasticum* in the experimental group were significantly higher than their counterparts in the control group (*p* < 0.01), whereas the content of methanogens in the experimental group was significantly lower than that of the control group (*p* < 0.01). However, the contents of cellulose-decomposing microbes, namely, *F. succinogenes*, *R. albus*, and *R. flavefaciens* exhibited no significant differences between the experimental group and the control group (*p* > 0.05). Taken together, the supplementation of DHP in the diet could alter the ruminal microbial communities in dairy cows.

### 3.7. DGGE Analysis of Microbe Communities in Rumen

**Figure 6:**
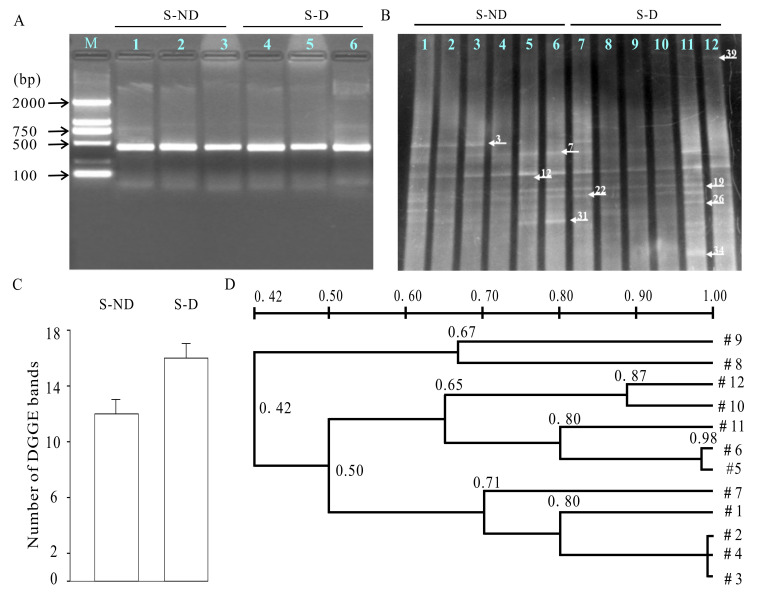
Effects of DHP on the ruminal bacterial community in dairy cows under HS conditions. (**A**) A representative agarose gel electrophoresis of rumen bacteria PCR products. Lanes 1-3 indicate electrophoresis results of PCR products from three different cows in the control group; lanes 4–6 indicate the electrophoresis results of three different cows in experimental group; M: DL2000 DNA marker. (**B**) Lanes 1–6 indicate the denaturing gradient gel electrophoresis (DGGE) results of PCR products from six different cows in the control group; lanes 7–12 indicate the DGGE results of PCR products from six different cows in the experimental group. Arrows indicate the bands of PCR products; (**C**) the number of DGGE bands in control group (S-ND) and the experimental group (S-D); (**D**) cluster analysis of the ruminal bacterial community between control group and experimental group.

The ruminal microbial communities were further detected by DGGE (Figure 6). The profiles of individual cows in the same treatment group were relatively similar. For example, the similarity between lane two and lane three (respectively representing an individual cow) was higher than 95% (Figure 6B), suggesting the similar composition of ruminal microbial community within the same group. However, the profiles greatly differed between the control group and the experimental group, although a small section of similarity was observed, indicating that the composition of the ruminal microbial community in the experimental group was different from that in the control group (Figure 6B,C). The bacteria derived from the rumen of dairy cows in control and experimental groups fell into mainly two clusters. The cluster derived from experimental group cows’ rumen fluids showed a low similarity of 42% with the other cluster, suggesting a remarkable alteration in microbial community induced by DHP (Figure 6D). In general, the addition of DHP induced an increase in the number of the dominant microbes, suggesting more complex microbial communities in the dairy cows treated by DHP.

Twenty-eight bands in the Figure 6C were retrieved. The cluster analysis of the ruminal bacterial community is shown in Figure 6D and Table 4. In the ruminal fluid samples, microbial communities fell into two main clusters. The DGGE profiles of cow eight and cow nine in the experimental group were clustered together, while the DGGE profiles of the other cows fell into the other cluster. The similarity between the two clusters was 42%. Meanwhile, the similarities between individual cows within the same group varied from 42% to 87%. After the amplification, cloning, and sequencing, thirty-two positive clones were selected from those dominant bands. These positive clones are highlighted with arrows in Figure 6B. After BLAST against the NCBI, the sequence information of these nine 16S rRNAs were submitted to Genbank. Their accession numbers were JF798509-JF798517 (Table 5). These data indicated that the addition of DHP to the diet drastically altered the ruminal microbial community in the dairy cows.

### 3.8. Phylogenic Tree Analysis

**Figure 7:**
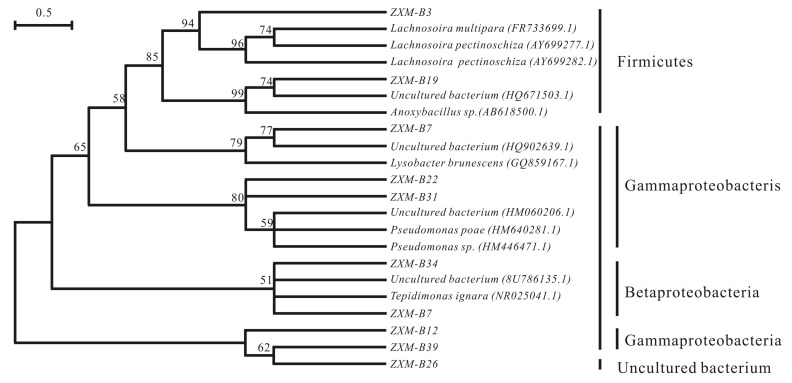
Phylogenic tree of rumen bacteria.

Based on the information of 16S rRNA sequences, a phylogeny tree was constructed using the neighbor-joining method (Figure 7). The nine dominant bacteria in ruminal fluids were identified, including Lachnospira pectinoschiza(AY699277.1), Lysobacter brunescens(GQ859167.1), Acinetobacter sp. JFAN2 (HQ693555.1), Anoxybacillus sp. IP-3 (AB618500.1), Pseudomonas sp. SUT 19 (HM446471.1), Pseudomonas sp. SUT 19 (HM446471.1), Tepidimonas aquatic (NR_025755.1), Xanthomonas axonopodis (AB101447.1), and a uncultured bacterium (EU843886.1) (Table 5).

## 4. Discussion

The present study mainly explored the effects of DHP addition to the diet on the antioxidant capacity and ruminal microbial communities in lactating dairy cows. The results demonstrated that DHP addition enhanced the antioxidant capacities both in the serum and rumen. Meanwhile, DHP addition altered the compositions of the ruminal microbial community in dairy cows. These data indicated that DHP addition to the diet helped to relieve the negative effects of HS on dairy cows. 

This experiment was carried out in the Yangtze River Dairy Co. Ltd. in Wuhu (Wuhan, China). In order to monitor HS, the temperature, humidity, and THI were analyzed in April and July (Figure 1). The results demonstrated that the minimal value of THI in July was 76.96, which exceeded the threshold of HS of 68.0 [26]. The level of HS to which the dairy cows were exposed was affected by effective temperature which included five environmental factors, namely, air temperature, humidity, air movement, solar radiation, and precipitation. However, only temperature and humidity were set as variables in the simple THI method which was effective and efficient in evaluating the effects of HS on dairy cows. For example, one previous study reported that the average daily milk yield decreased approximately 0.2 kg per unit increase in THI, when THI ≥ 72 [27]. The high THI values in July observed in this experiment suggest that the dairy cows were possibly under HS conditions. 

In the hot and humid summer, both the rapid increase in temperature and humidity resulted in the occurrence of HS, which can decrease milk yield in dairy cows. The decreased milking performance was partly due to the reduction in the antioxidant capacity of dairy cows. Therefore, we first analyzed the correlation between the most commonly used antioxidant indexes and the decreased rate of milk yield. The correlation analysis suggested that SOD, GSH-Px, T-AOC (*p* < 0.01), and MDA (*p* < 0.05) showed significant correlation with the reduced milking ability (Table 3). These four correlated indexes were further analyzed to determine the effects of DHP on dairy cows. In addition, twenty heat-sensitive cows were selected and used in the subsequent experiment. 

Daily metabolic activities in dairy cows produce a lot of free radicals and other bioactive substances [5]. Under HS conditions, the antioxidant ability of the blood in the dairy cows is decreased, while the expression of inflammation factors is increased. These changes will result in tissue damage and inflammation [1]. As shown in Figure 3 and Figure 4, the addition of DHP significantly increased the activities of SOD, GSH-Px in blood and ruminal fluids and the activities of T-AOC in ruminal fluids (*p* < 0.01). Our data demonstrated that DHP addition in the diet improved the antioxidant capacity in dairy cows. Furthermore, the significantly decreased MDA level in the experimental group indicated that DHP addition in the diet decreased the toxic metabolic products in the dairy cows (*p* < 0.01). Blood samples collected before the start of the experiment using as a covariant during both non-HS and HS seasons will enhance the scientificity and rigorousness of the experimental design. Taken together, the results indicate that the addition of DHP in the diet can partly reverse the harmful effects of HS on the dairy cows. 

In the rumen, a high anaerobic environment, the microbes are more sensitive to free radicals and oxidative stresses induced by external environment changes. Wang et al. in 2010 reported that unsaturated fatty acids in the diet led to oxidative stress which was harmful to the growth of rumen cellulolytic bacteria [28]. This harmful effect can be reversed by antioxidants such as vitamin E which can also improve the stability of high-fat feed [29]. This report is in line with our observations of antioxidant capacities in the sera and ruminal fluids of DHP-treated dairy cows (Figure 2 and Figure 3). Similarly, Vazquez et al. (2008) reported that feeding Agrado Plus, a type of mixed antioxidant, improved lactation performance and the antioxidant status of the cow [30]. However, the responses of ruminal microbes to different substances such as fat, Agrado Plus, and DHP differed depending on many variables. However, the underlying mechanisms of these responses were unclear and remained to be further investigated.

Stresses were harmful to rumen fermentation and the growth of rumen microbes [31]. HS significantly resulted in the decrease in most types of rumen microbes such as Methanobacteria, *R. albus*, *F. succinogenes* and fungi. In our study, DHP treatment resulted in the occurrence of some new bands and the disappearance of some bands, but the total number of bands was increased (Figure 6). Our results demonstrated that the decrease in rumen microbes could be reversed by DHP addition, suggesting that DHP altered the structure and component of rumen bacteria in dairy cows. Unexpectedly, the amount of the Methanobacteria decreased after DHP treatment, which might be due to the reduction in two types of methane-synthesizing materials (acetic acid and hydrogen). On the one hand, the content of acetic acid decreased under HS conditions. On the other hand, DHP increased the number of hydrogen-related microorganisms such as *B. fibrisolvens* and *C.proteoclasticum*, which consumed a lot of hydrogen. Therefore, the growth of Methanobacteria was inhibited, thus leading to the reduced methane production [32]. These data suggested that DHP could significantly alter the structure and component of rumen microbes, in turn affecting the fermentation mode in the rumen of dairy cows.

Many rumen bacteria can be analyzed only by using molecular biological techniques such as RT-PCR and DGGE due to their specific nutritional requirements. Our phylogenetic tree results of rumen bacteria demonstrated that most rumen bacteria belonged to Proteobacteria and Firmicutes. Some bacteria such as band three *Lachnospira* mainly appeared in the control group, while other bacteria such as band seven *Xanthomonadaceae* mainly appeared in the experimental group. In addition, some bacteria such as band 12 Acinetobacter, band 19 *Anoxybacillus*, and band 26 an unknown bacterium could be detected with or without DHP. Taken together, DHP could enhance the diversity of rumen bacteria. Particularly, it can promote the growth of bacteria *Xanthomonadaceae* and *Xanthomonas*. In summary, DHP addition in the diet is helpful for improving the ruminal microbe biodiversity and increasing the antioxidant capabilities of dairy cows under HS conditions. These beneficial effects can improve the health status of dairy cows, in turn facilitating an increase in milk yield. However, the effects of DHP on the metabolism, blood physiochemical characteristics, and physiological connections among these antioxidant indexes remain to be further investigated.

## 5. Conclusions

Addition of DHP in lactating dairy cow diet enhances the antioxidative capacity and improves composition of dairy cow ruminal microbiota under HS. DHP additives may be a useful way to rescue dairy cows from HS, which would benefit the dairy cow industry.

## Figures and Tables

**Table 1:** Formulation and nutritional ingredients of standard diet.

Diet Components	Formulation (%)	Nutritional Ingredients	Content (%)
Corn silage	55.6	Dry matter (DM, %)	51.4
Peanut hay	13.9	Dry matter intake (DMI, kg/d)	18.5
Corn	15.6	Net energy for lactation (NEL, MJ/d)	29.7
Wheat bran	6.1	Crude protein (CP, %)	15.37
Soybean meal	4.6	Acid detergent fiber (ADF, %)	24.09
Cotton seed meal	2.4	Neutral detergent fiber (NDF, %)	37.42
CaHPO_4_	0.6	Calcium (Ca, %)	0.91
NaHCO_3_	0.3	Phosphorus (P, %)	0.48
Premix	0.9		
Total	100.0		

Note: Nutritional ingredients were based on the content of DM. The contents of CP, ADF, DNF, Ca and P were measured, while the content of NEL was calculated.

**Table 2:** Primers for real-time PCR assay (used in this study).

Target Species	Primer	Sequence (5′-3′)	Reference
Total bacteria	F R	CGGCAACGAGCGCAACC CCATTGTAGCACGTGTGTAGCC	[19]
Fungi	F R	GAGGAAGTAAAAGTCGTAACAAGGTTTC CAAATTCACAAAGGGTAGGATGATT	[19]
Protozoa	F R	GCTTTCGWTGGTAGTGTATT CTTGCCCTCYAATCGTWCT	[20]
Methanogens	F R	TTCGGTGGATCDCARAGRGC GBARGTCGWAWCCGTAGAATCC	[20]
*B. fibrisolvens*	F R	ACACACCGCCCGTCACA TCCTTACGGTTGGGTCACAGA	
*C.proteoclasticum*	F R	TCCGGTGGTATGAGATGGGC GTCGCTGCATCAGAGTTTCCT	
*F. succinogenes*	F R	GTTCGGAATTACTGGGCGTAAA CGCCTGCCCCTGAACTATC	[21]
*R. albus*	F R	CGGCAACGAGCGCAACCC CCATTGTAGCACGTGTGTAGCC	[22]
*R. flavefaciens*	F R	CGAACGGAGATAATTTGAGTTTACTTAGG CGGTCTCTGTATGTTATGAGGTATTACC	[23]

**Table 3:** Correlation analysis between serum antioxidant capacity and the milk yield.

Items	Milk Yield Decrease	
	Correlation Coefficient (r Values)	*p* Values
SOD (U/mL)	−0.462	*p* < 0.01
GSH-Px (U/mL)	−0.619	*p* < 0.01
CAT (U/mL)	−0.291	*p* > 0.05
T-AOC (U/mL)	−0.515	*p* < 0.01
MDA (nmol/mL)	0.371	*p* < 0.05

**Table 4:** Similarities in rumen bacterial community between control group and experimental group.

Lane	1	2	3	4	5	6	7	8	9	10	11	12
1	100.0	80.1	79.8	80.3	69.2	70.8	60.5	40.1	20.0	19.2	52.9	32.7
2	80.1	100.0	99.3	99.3	68.2	68.4	74.8	50.1	24.9	24.0	62.4	40.3
3	79.8	99.3	100.0	99.3	68.3	68.5	74.2	50.0	24.9	24.0	62.5	40.4
4	80.3	99.3	99.3	100.0	68.1	68.3	74.7	49.6	24.8	23.9	62.3	40.2
5	69.2	68.2	68.3	68.1	100.0	98.4	50.8	50.6	34.3	55.1	80.4	67.6
6	70.8	68.4	68.5	68.3	98.4	100.0	50.9	50.7	34.3	55.0	80.3	67.5
7	60.5	74.8	74.2	74.7	50.8	50.9	100.0	49.8	24.8	23.9	46.4	40.2
8	40.1	50.1	50.0	49.6	50.6	50.7	49.8	100.0	67.2	47.7	47.1	41.0
9	20.0	24.9	24.9	24.8	34.3	34.3	24.8	67.2	100.0	72.1	46.9	61.6
10	19.2	24.0	24.0	23.9	55.1	55.0	23.9	47.7	72.1	100.0	66.6	87.2
11	52.9	62.4	62.5	62.3	80.4	80.3	46.4	47.1	46.9	66.6	100.0	77.4
12	32.7	40.3	40.4	40.2	67.6	67.5	40.2	41.0	61.6	87.2	77.4	100.0

Note: lanes 1–6 mean the DGGE profiles of six cows from control group; lane 7–12 mean the DGGE profiles of six cows from experimental group.

**Table 5:** Detailed information of rumen bacterial 16S rDNA clones.

Bands	Closest Known Species	Sequence Similarity	Length (bp)	Taxonomic Group	Acc. Number
B3	Lachnospira pectinoschiza AY699277.1	99%	433	Bacteria; Firmicutes; Clostridia; Clostridiales; Lachnospiraceae; Lachnospira.	JF798509
B7	Lysobacter brunescens GQ859167.1	99%	434	Bacteria; Proteobacteria; Gammaproteobacteria; Xanthomonadales; Xanthomonadaceae; Lysobacter.	JF798510
B12	Acinetobacter sp. JFAN2 HQ693555.1	99%	435	Bacteria; Proteobacteria; Gammaproteobacteria; Pseudomonadales; Moraxellaceae; Acinetobacter.	JF798511
B19	Anoxybacillus sp. IP-3 AB618500.1	99%	436	Bacteria; Firmicutes; Bacillales; Bacillaceae; Anoxybacillus.	JF798512
B22	Pseudomonas sp. SUT 19 HM446471.1	99%	434	Bacteria; Proteobacteria; Gammaproteobacteria; Pseudomonadales; Pseudomonadaceae; Pseudomonas.	JF798513
B26	Uncultured bacterium EU843886.1	99%	437	Bacteria; environmental samples	JF798514
B31	Pseudomonas sp. SUT 19 HM446471.1	99%	434	Bacteria; Proteobacteria; Gammaproteobacteria; Pseudomonadales; Pseudomonadaceae; Pseudomonas.	JF798515
B34	Tepidimonas aquatica NR_025755.1	99%	430	Bacteria; Proteobacteria; Betaproteobacteria; Burkholderiales; Tepidimonas.	JF798516
B39	Xanthomonas axonopodis AB101447.1	99%	433	Bacteria; Proteobacteria; Gammaproteobacteria; Xanthomonadales; Xanthomonadaceae; Xanthomonas.	JF798517
